# Dual‐Functional Membranes With Interfacial MXene/PFSA Nanoarchitectures for Low Hydrogen Permeation in Water Electrolysis and Reduced Vanadium Ion Crossover in Flow Batteries

**DOI:** 10.1002/advs.78270

**Published:** 2026-10-14

**Authors:** Panpan Guan, Mingjia Jiang, Yunhu Jiang, Wei Li, Yecheng Zou, Lixiao Zhang, Kaifeng Long, Ziqi Sun, Ting Liao, Hua Kun Liu, Ding Yuan, Dingsheng Wang, Shi Xue Dou, Yuhai Dou

**Affiliations:** ^1^ Institute of Energy Materials Science (IEMS) University of Shanghai for Science and Technology Shanghai China; ^2^ Shandong Key Laboratory of Core Materials and Technologies for Hydrogen Energy and Dongyue Future Hydrogen Energy Materials Company Zibo China; ^3^ School of Chemistry and Physics Queensland University of Technology Brisbane Australia; ^4^ Faculty of Engineering School of Mech. Medical & Process Engineering Queensland University of Technology Brisbane Australia; ^5^ Engineering Research Center of Advanced Rare Earth Materials Department of Chemistry Tsinghua University Beijing China

**Keywords:** 2D MXene, composite membranes, hydrogen permeation and vanadium ion crossover, proton exchange membrane water electrolysis, vanadium redox flow batteries

## Abstract

Proton exchange membrane water electrolysis (PEMWE) and vanadium redox flow batteries (VRFBs) suffer from hydrogen and vanadium ion crossover when using conventional perfluorosulfonic acid (PFSA) membranes. Here we report a layer‐by‐layer MXene/PFSA membrane architecture in which MXene nanosheets are integrated into PFSA coating layers to create MXene‐regulated nanointerfaces that decouple proton conductivity from gas and ion permeation. The lamellar MXene framework introduces tortuous transport pathways and selectively perturbs PFSA hydrophilic domain organization while preserving continuous proton‐conducting networks through interfacial hydrogen bonds. As a result, the optimized membrane exhibits simultaneously enhanced H^+^/H_2_ and H^+^/VO^2+^ selectivity, achieving up to 43.0% lower hydrogen permeability and 37.5% reduced vanadium ion diffusion compared to pristine PFSA, without sacrificing proton conductivity. It exhibits stable long‐term operation in PEMWE with lower hydrogen and retains 67.0% capacity in VRFB over 470 cycles, outperforming pristine PFSA more than 40% of capacity retention. Small‐ and wide‐angle X‐ray scattering reveal MXene‐induced disordering and expansion of large‐scale hydrophilic domains, while density functional theory (DFT) calculations reveal that the MXene/PFSA interface selectively increases transport energy barriers for hydrogen and vanadium ions while preserving low barriers for proton migration. These results validate a versatile interfacial strategy toward overcoming selectivity limitations for both PEMWE and VRFB.

## Graphical abstract

A PFSA/MXene composite membrane is developed for both PEMWE and VRFB applications. MXene incorporation simultaneously enhances proton conductivity and suppresses hydrogen/vanadium crossover. The interaction between MXene functional groups and PFSA's –SO_3_H reduces the proton diffusion barrier via hydrogen‐bonding networks, while the disordered hydrophilic channels increase tortuosity for larger species. The composite membrane exhibits excellent cell performance and stability in both systems.

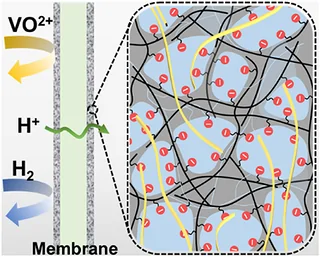

## 1 Introduction

The global transition toward carbon neutrality urgently requires the deployment of renewable energy sources such as solar and wind power [1]. While their inherent fluctuations and intermittency create mismatches between energy supply and grid demand, necessitating complementary systems for both long‐term energy carriers (e.g., green hydrogen via water electrolysis) [2, 3, 4] and energy storage (e.g., flow batteries) [5]. Proton exchange membrane water electrolysis (PEMWE), renowned for its high efficiency and rapid response, splits water into hydrogen and oxygen by the membrane electrode assembly (MEA) [6], where proton exchange membrane (PEM) facilitates efficient proton (H^+^) transport while blocking hydrogen crossover [7]. Minimizing hydrogen permeation through the PEM is crucial for PEMWE, especially when operating under differential pressure conditions. Similarly, the all‐vanadium redox flow battery (VRFB), has emerged as a promising solution for large‐scale energy storage systems, benefiting from decoupled energy/power capacity, scalable design, and recyclability [8, 9]. In VRFBs, the PEM facilitates proton transport while preventing cross‐mixing of the V^2+^/V^3+^ (anolyte) and VO^2^^+^/VO_2_^+^ (catholyte) redox couples. However, vanadium crossover through perfluorosulfonic acid (PFSA) membranes causes electrolyte contamination and capacity decay, severely compromising Coulombic efficiency (CE). Central to both technologies are PEM, which face similar yet application‐specific challenges. As the state‐of‐the‐art PEM, the PFSA membranes are widely applied in PEMWEs and VRFBs due to their unique nanophase‐segregated morphology, featuring interconnected hydrophilic ionic channels with typical distances of 2–3 nm and 10−15 nm, embedded within a semicrystalline PTFE matrix [10, 11, 12]. However, the hydrophilic domains not only enable rapid proton transport but also serve as conduits for hydrogen permeation in PEMWE and vanadium cation migration in VRFBs. In PEMWE, hydrogen (kinetic diameter: 0.289 nm) [13] predominantly permeates through PFSA PEM via the dissolution‐diffusion mechanism, wherein diffusion through hydrophilic channels is primarily governed [14]. In VRFBs, the vanadium cations transportation in PEM is primarily through the hydrophilic channels, driven by the size sieving effect and the Donnan effect. In sulfuric acid electrolytes, vanadium ions (VO^2+^) have a hydrated radius above 0.6 nm, compared to ∼0.24 nm for H^+^ [15, 16, 17]. Both scenarios highlight a unified scientific challenge: reconciling proton conduction with selective ion exclusion, specifically enhancing the selectivity of H^+^/H_2_ and H^+^/V^n+^.

There remains a pressing demand for advanced PEMs that meet the stringent requirements of these electrochemical systems. 2D materials, such as graphene oxide (GO), boron nitride (BN), molybdenum disulfide (MoS_2_), etc., have been integrated into PEMs to modify PEM properties, due to their extensive specific surface area, rich functional groups, and superior mechanical properties. For hydrogen blocking, hexagonal boron nitride (h‐BN) was spin‐coated [18] or hot‐pressed [19] on the surface of Nafion membrane to form the gas barrier layer, resulting in 27%−42% lower hydrogen permeability but lower proton conductivity [19]. Similarly, graphene, BN, and MoS_2_ barrier layers on Nafion211 achieved 4.4%−46.1% hydrogen crossover reduction accompanied by over 20% drop in peak power density [20]. The carbon quantum dots (CQDs) [21] and MXene/CeO_2_ [22] on Nafion membrane reduced hydrogen permeation by 11.4% and 23.8%, respectively. Besides these, the graphene and BN were also sandwiched within the Nafion membrane to construct a multi‐layer structure, with the result that most hydrogen crossover reduction was below 50% compared with the pristine membrane [23, 24, 25, 26].

In VRFBs, hydrocarbon‐based polymers (e.g., sulfonated poly (ether ether ketone), sPEEK) exhibit reduced vanadium ion permeability due to their less continuous channels than PFSA membranes [27]. Moreover, polymer blending has also been employed to enhance vanadium rejection by tailoring phase‐separated morphologies [28, 29]. Leveraging the size disparity between hydrated vanadium ions (>0.6 nm) [16] and protons (<0.24 nm) [17, 30], nanofillers such as zeolites [30] and covalent organic framework (COFs) [31], enable precise regulation of hydrophilic pore dimensions, achieving synergistic optimization of proton conductivity and vanadium blocking. In contrast, the exploration of 2D materials for vanadium ion blocking remains relatively limited, with graphene‐based nanomaterials being the most studied. Y. Hwan et al. coated Nafion117 with a 4−5 µm GO layer, reducing vanadium permeability from 20.5 × 10^−7^ to 6.1 × 10^−7^ cm^2^ min^−1^ while improving CE and energy efficiency (EE) [32]. G. He et al. further aligned a 400−440 nm ultrathin GO nanosheet layer on PFSA, suppressing vanadium permeation by two orders of magnitude versus Nafion212 and maintaining proton conductivity for balanced performance [33]. GO is now often combined with other functional particles (e.g. WO_3_‐decorated GO nanosheets [34] or GO/Schiff base network type COFs (GO/SNW) hybrid [35]) to block the vanadium ions. Beyond PFSA, GO has been incorporated into various polymer matrices, such as sPEEK, to reduce VO^2+^ crossover while maintaining proton conductivity [36, 37]. Other 2D materials such as C_3_N_4_, MoS_2_, and oxidized black phosphorus (O‐bPn) [38] also demonstrate vanadium‐blocking potentials.

Overcoming the selectivity–conductivity trade‐off requires precise nanoscale control of transport without disrupting the intrinsic PFSA channel network. 2D materials, particularly MXenes, are promising due to their atomic thickness, layered structure, and abundant surface functional groups (e.g., −O−, −F, −OH) that enable hydrogen bonding with the polymer matrix to facilitate proton transfer while providing size‐sieving effects for ion selectivity. Recent studies have shown that MXenes can enhance ion transport or mechanical stability in polymer membranes, yet their role in reorganizing PFSA ionic domains and selectively regulating molecular versus ionic transport remains poorly understood. Moreover, MXene‐based PEM modifications have largely focused on single‐device applications, leaving their broader relevance to multiple electrochemical systems unexplored. In this work, we introduce a layer‐by‐layer MXene/PFSA membrane architecture where MXene/PFSA composite layers are coated on both sides of a PFSA core membrane, confining MXene to interfacial regions where selectivity is most critical while preserving the bulk proton‐conducting network of PFSA. To the best of our knowledge, this is the first MXene‐coated PFSA membrane designed for simultaneous suppressing H_2_ and VO^2+^ crossover while enhancing proton conductivity. A comprehensive literature survey (summarized in Tables S1 and S2) reveals that studies on MXene‐modified proton exchange membranes for PEMWE or VRFB remain limited, and no previous report has extended the same MXene/PFSA membrane to both applications. Moreover, the layer‐by‐layer coating architecture adopted in this work has not been reported elsewhere for dual‐function purposes as well. Notably, compared with the MXene‐based membranes documented in the literature, our PFSA/MXene composite membrane exhibits competitive or superior performance in both systems. Therefore, this work not only provides a viable strategy for overcoming the selectivity–conductivity trade‐off in PFSA membranes but also expands the application scope of MXene‐based materials across diverse electrochemical energy technologies.

## 2 Results and Discussions

### 2.1 MXene Preparation

The Ti_3_C_2_T_x_ (MXene) filler was synthesized through selective etching of aluminum (Al) layers from Ti_3_AlC_2_ (MAX) using a LiF/HCl solution, accompanied by subsequent centrifugal washing and ultrasonic exfoliation to ultimately yield few‐layer MXene structures, as described in Figure 1a. Morphological evolution was investigated through scanning electron microscope (SEM) imaging (Figure 1b–d). The dense MAX precursor (Figure 1b) transforms into an accordion‐like layered structure Ti_3_C_2_T_x_ (Figure 1c), with subsequent sonication producing few‐layer Ti_3_C_2_T_x_ flakes (Figure 1d). The X‐ray diffraction (XRD) analysis was conducted to compare the structural change before and after etching (Figure 1e). Notably, the (002) diffraction peak exhibits a 2θ shift from 9.7° to 6.3° in MXene, indicating an interlayer spacing change from 0.91 to 1.40 nm according to Bragg's Equation. This confirms the lattice expansion along the c‐axis [39]. Moreover, complete elimination of the characteristic (104) peak at 39° verifies successful aluminum removal and MXene formation [39]. Complementary X‐ray photoelectron spectroscopy (XPS) spectrum (Figure 1f) reveals elemental composition and bonding states, with distinct O 1s and F 1s signals confirming Ti−O and Ti−F existence. High‐resolution Ti 2p spectra (Figure 1g) deconvolute into bonding configurations related to Ti−O/Ti═O (455.6 eV/459.1 eV), Ti−C (454.9 eV), and Ti−F (456.7 eV) [40, 41]. Figure S1a further resolves oxygen environments into O = Ti (530.0 eV/531.5 eV) and O−H groups (532.7 eV) [42], while fluorine analysis (Figure S1b) identifies Ti−F bonding at 685.4 eV [43]. The C 1s spectra (Figure S1c) were fitted with components related to C−Ti (281.8 eV and 282.3 eV) [44], C−C (284.8 eV), and C−O (286.2 and 288.1 eV) [45]. These findings collectively demonstrate surface termination with −O, −OH, and −F moieties, consistent with established MXene characteristics and confirming effective delamination and successful MXene synthesis.

**FIGURE 1:**
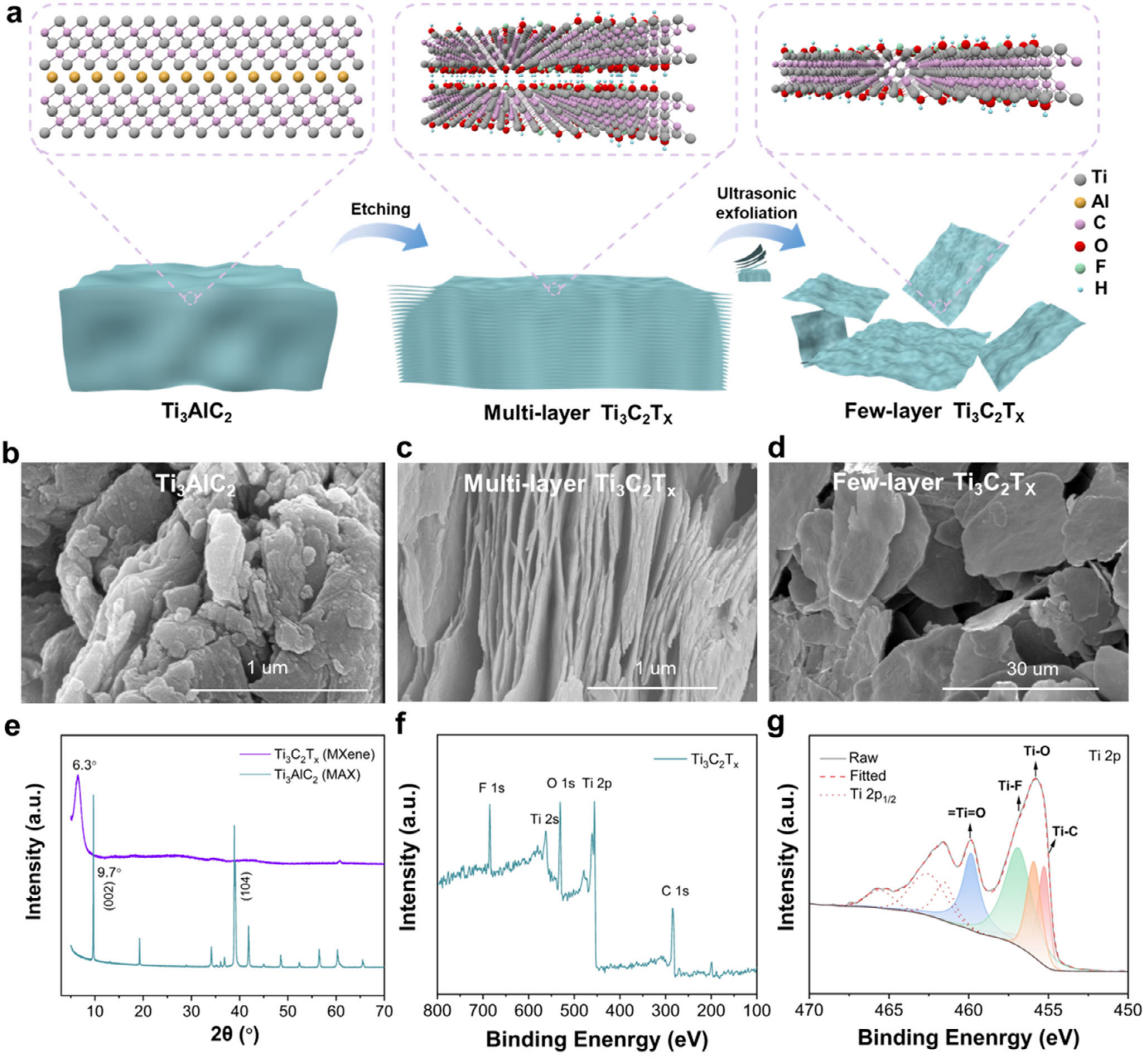
(a) Schematic illustration for the synthesis of few‐layer Ti_3_C_2_T_x_; SEM images of (b) Ti_3_AlC_2_, (c) multi‐layer Ti_3_C_2_T_x_, and (d) few‐layer Ti_3_C_2_T_x_; (e) XRD patterns of Ti_3_AlC_2_ and Ti_3_C_2_T_x_; (f) XPS spectrum and (g) high‐resolution XPS spectra of Ti 2p for the few‐layer Ti_3_C_2_T_x_.

**FIGURE 2:**
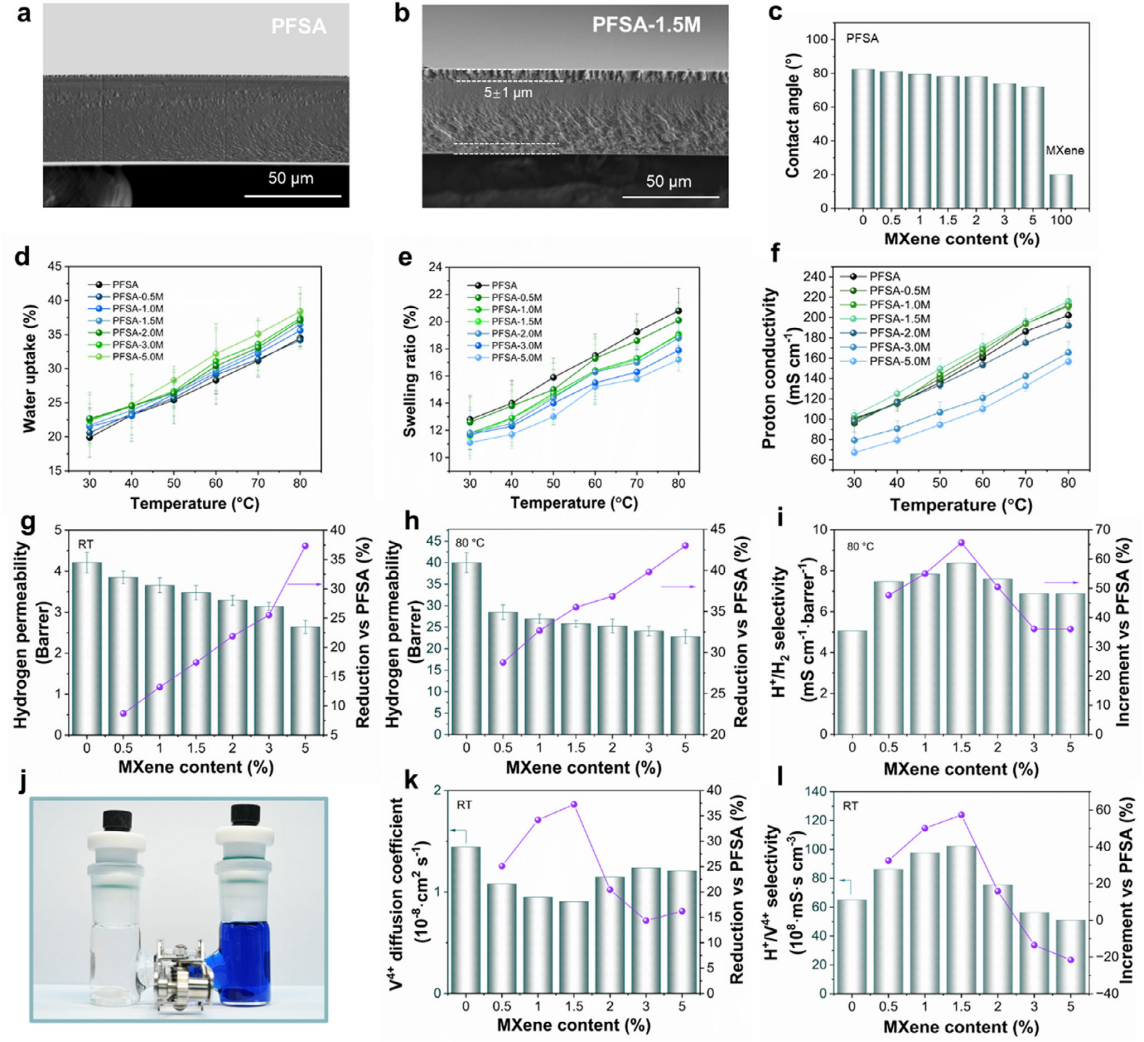
Cross‐section SEM images of (a) PFSA and (b) PFSA‐1.5 M membrane; (c) water contact angles of membranes and MXene; (d) water uptake, (e) swelling ratio, and (f) proton conductivity of MXene/PFSA membranes. Hydrogen permeability at (g) RT and (h) 80°C, and (i) proton selectivity (H^+^/H_2_) at 80°C of MXene/PFSA membranes; (j) H‐type diffusion cell for VO^2+^ permeation test; (k) V^4+^ diffusion coefficient and (l) H^+^/V^4+^ selectivity for MXene/PFSA membranes.

### 2.2 Membrane Fabrication, Hydrogen Permeation, and Vanadium Crossover

The MXene‐coated PFSA membranes were fabricated via a solution casting method. The functional layers with varying concentrations of MXene (0.5–5.0 wt.%) dispersed in PFSA were sequentially coated on both sides of the PFSA membrane, forming a sandwich‐like layered architecture, as illustrated in Figure S2. The resultant MXene‐coated PFSA membranes were systematically designated as PFSA‐xM, where “x” denotes the MXene mass percentage relative to the PFSA matrix in the coating layers. By controlling the coating thickness, the MXene/PFSA layers on both sides of the PFSA membrane were maintained at 5 ± 1 µm, while the whole membrane thickness was maintained at 50 ± 2 µm, as demonstrated by the cross‐sectional SEM images of the membranes (Figure 2a,b and Figure S3). We selected 5 µm as the optimal coating thickness for the final membrane design, as it offers a balanced overall performance with acceptable proton conductivity and effective hydrogen barrier properties (Figure S4). The morphology of SEM reveals robust interfacial adhesion between the MXene/PFSA coating layer and the PFSA matrix. It is worth noting that the cross‐sectional morphologies of the top and bottom coating layers appear slightly different under SEM, which may be related to the distinct drying conditions at the two surfaces during membrane fabrication, and the origin of this phenomenon warrants further investigation. Ion exchange capacity (IEC) of the composite membrane decreases slightly with increasing MXene content (Figure S5), which is attributed to hydrogen bonding between MXene (─OH, ─O, ─F) and ─SO_3_H groups (partially consumes acidic protons).

Given that the hydrophilicity of PFSA membranes is essential for achieving high proton conductivity and surface wettability facilitates interfacial compatibility with catalytic layers, water contact angle measurements were conducted to evaluate hydrophilic modifications in the composite membranes. As shown in Figure 2c and Figure S6, the pristine PFSA membrane exhibits a contact angle of ∼82.5°, while the MXene counterpart demonstrates a significantly reduced angle of ∼20.0°. A reduction in contact angle from 81.0° to 72.0° was observed for the MXene‐coated PFSA membranes as the MXene loading increased from 0.5 to 5.0 wt.%. This trend indicates enhanced surface hydrophilicity with increasing MXene content, attributed to the water‐affinity of the abundant functional groups, confirming the improved membrane's wettability.

While excessive water uptake typically induces membrane swelling and compromises PEM's mechanical stability, the composite membranes with MXene/PFSA coating layers exhibit an exceptional balance of elevated hydration with moderated dimensional expansion. The data in Figure 2d,e reveal a consistent temperature‐driven augmentation in both water uptake and swelling ratio for all membranes. For water uptake, MXene‐coated membranes are consistently higher than the pristine PFSA membrane. At 30°C, water uptake rises from 19.9% (PFSA) to 22.7% (PFSA‐5.0 M), escalating further to 34.5% and 37.1%, respectively, at 80°C. This higher water uptake of composite membranes correlates directly with MXene concentration, attributed to the hydrophilic groups, such as −OH, on MXene surfaces, increasing hydrophilic domains [46, 47]. The composite membranes display reduced swelling ratios compared to the pristine PFSA membrane, with further suppression observed at higher MXene loadings. This optimal balance makes it well‐suited for membrane applications. This inhibition effect is attributable to the enhanced mechanical robustness from the coating layers incorporating inorganic 2D MXene in PFSA matrix. As shown in Figure S7, the mechanical strength increases from 18.6 MPa for the PFSA membrane to 30.4 MPa for the PFSA‐1.5 M membrane. Meanwhile, the elongation at break decreases from 262.1% to 192.3% due to the enhanced rigidity. The results align well with previously reported MXene reinforcement mechanisms in anion‐exchange membranes [48, 49].

The solid ^1^H NMR spectra (Figure S8a) show two distinct regions: a peak at 2–3 ppm (residual water/solvent) and a peak at 7–9 ppm (acidic protons of ─SO_3_H groups) [50]. In general, a higher acidic proton concentration leads to a downfield shift. Compared with pristine PFSA, the acidic proton peak of PFSA‐1.5 M shifts to lower chemical shift (upfield), indicating reduced acidic proton concentration due to hydrogen bonding between MXene (─OH, ─O, ─F) and ─SO_3_H groups. Additionally, the slightly narrower peak width for PFSA‐1.5 M suggests enhanced proton mobility, correlated with the hydrogen‐bonding network formed between MXene and PFSA. The T_2_ relaxation curves (Figure S8b) reveal two distinct water populations: bound water (short T_2_, tightly associated with ─SO_3_H groups) and free water (long T_2_, more mobile) [51]. The bound water fraction in PFSA‐1.5 M is 29.5%, lower than that of pristine PFSA (35.4%), due to hydrogen‐bonding interactions between MXene and –SO_3_H groups altering water–polymer interactions. Notably, both T_2_ peaks in PFSA‐1.5 M shift to slightly longer relaxation times, indicating less restricted proton mobility, which is attributed to the more extensive hydrogen‐bonding network facilitating proton transfer. The coexistence of bound and free water confirms that proton transport occurs via both Grotthuss and vehicular mechanisms.

The proton conductivity of the composite membranes, decisive to cell performance, was measured under fully hydration conditions across a temperature range of 30°C−80°C. Figure 2f reveals a non‐monotonic relationship between proton conductivity and MXene loading, with the PFSA‐1.5 M membrane (1.5 wt.% MXene) achieving optimal conductivity of 215.9 mS cm^−1^ at 80°C─a 6.7% enhancement over pristine PFSA. This could be attributed to the hydrophilic functionalities on MXene surfaces, which enhance membrane hydrophilicity, facilitating proton transport via the vehicle mechanism. The formation of hydrogen bonding by oxygen‐containing groups establishes hydrogen‐bond networks and promotes proton hopping through the Grotthuss mechanism [46, 52]. Furthermore, the lamellar architecture enables the formation of extended proton transfer pathways along interfacial domains [22]. To clarify the proton conduction mechanism, the activation energy (*E_a_*) was calculated (Figure S9). The pristine PFSA membrane shows an *E_a_* of 12.94 kJ mol^−1^, and the composite membrane shows the *E_a_* of 12.07–15.07 kJ mol^−1^. These values confirm that the Grotthuss mechanism is the dominant proton transport pathway in both membranes. When the MXene content is above 2 wt.%, the proton conductivity obviously decreases, which could be caused by the aggregation of MXene, hampering the formation of uninterrupted proton transport pathways [22]. The results of thermogravimetric (TG) analysis also indicate the good water absorption capability of the composite membrane, since the water‐related mass loss between 30°C and 200°C for composite membranes is larger than the pristine PFSA membrane (Figure S10) [10]. The weight loss that occurs between 290°C and 310°C is due to the loss of sulfonic acid groups on the side chains of PFSA, and the decomposition near 440°C is related to the breakdown of the fluorocarbon backbone chains of the polymer matrix [53]. The rapid mass loss of the composite membranes within 290°C−310°C is likely attributed to MXene‐induced disruption of PFSA's microstructural phase separation (e.g., ionic cluster rearrangement), accelerating decomposition, rather than oxygen‐containing functional groups degradation on MXene surfaces, since the low MXene content and absence of significant mass loss in MXene's TG profile within this temperature range. Crucially, the incorporation of MXene exerts a negligible impact on the thermal decomposition temperature of the PFSA matrix, confirming preserved thermal stability.

The hydrogen permeability of the membranes was evaluated ex situ using the pressure differential method. The hydrogen permeability was determined through real‐time pressure sensor monitoring of permeated hydrogen flux under a transmembrane pressure gradient established by supplying 1 atm H_2_ to the feed side while maintaining the permeate side close to vacuum (<20 Pa). The hydrogen permeability at room temperature (RT) exhibits a progressive decline with elevated MXene content in the coating layer (8.7%–37.3% reduction versus pristine PFSA), as shown in Figure 2g. Elevated temperatures intensify hydrogen molecular motion and enhance hydrogen diffusion coefficients, leading to increased hydrogen permeability. At 80°C, the hydrogen permeability of pristine PFSA reaches up to 40 Barrer compared to < 4.5 Barrer at RT, while the composite membranes demonstrate 28.8%−43.0% lower values (Figure 2h). These results confirm the hydrogen‐blocking capability of the MXene/PFSA coating layer. Due to the mutually restrictive relationship between hydrogen permeation and proton conduction, the H^+^/H_2_ selectivity at 80°C is employed to assess ion selectivity. As shown in Figure 2i, the best H^+^/H_2_ selectivity is achieved with the PFSA‐1.5 M membrane, which is 65.6% higher than the PFSA, indicating the optimal balance between proton conductivity and hydrogen permeation.

In addition to hydrogen permeation, the crossover of vanadium ions through PFSA membranes poses a significant challenge that severely compromises both CE and EE of VRFBs. Considering that both vanadium ions and hydrogen permeation predominantly rely on the nano‐scale phase‐separated hydrophilic channels within PFSA membranes, and the notably larger ionic size of vanadium ions (>0.6 nm [16]) compared to hydrogen (0.289 nm [13]), the design of MXene‐coated PFSA membranes aimed to impede vanadium ion permeation as well. The mitigation of vanadium ion crossover is critical to preserving capacity retention and operational longevity in VRFBs, necessitating membranes with suppressed vanadium permeability. Although the permeability hierarchy follows V^2+^ > VO^2+^ > VO_2_^+^ > V^3+^, VO^2+^ concentration remains the primary analyte for assessing vanadium ion crossover due to the susceptibility of V^2+^ to aerial oxidation, which complicates its experimental quantification [36, 54]. The RT VO^2+^ permeation behavior of the membranes was evaluated in an H‐type diffusion cell (Figure 2j), with permeated vanadium ion concentrations quantified by UV–vis spectroscopy (Figures S11 and S12). Figure 2k illustrates the temporal evolution of VO^2+^ diffusion coefficient (D) for each membrane, revealing suppressed vanadium ion permeation with increasing MXene content to 1.5 wt.%, then slight increases when MXene content is higher than 2.0 wt.%. The PFSA‐1.5 M membrane displays a 37.5% lower VO^2+^ diffusion coefficient (0.90 × 10^−8^ cm^2^ s^−1^) compared to the pristine PFSA membrane (1.44 × 10^−8^ cm^2^ s^−1^). The calculated ion selectivity of H^+^/ VO^2+^ is shown in Figure 2l, with the result of PFSA‐1.5 M showing a 57.4% higher selectivity than that of PFSA. The high vanadium ion resistance results from the blocking function of MXene to vanadium ions, primarily by the size exclusion mechanism [55]. Meanwhile, the cation‐selective adsorption of Ti sites on MXene (negatively charged MXene) enables vanadium sieving, promoting proton‐selective transport while effectively inhibiting vanadium crossover by the Donnan effect [56]. When the MXene content is higher than 2.0 wt.%, the agglomeration of MXene may lead to charge shielding, which reduces the Donnan effect and results in a slight increase in the permeation of vanadium ions, since the permeation of vanadium ions is more driven by the concentration gradient.

### 2.3 PEMWE Performance and Durability

The comprehensive ex situ results demonstrate the MXene‐coated PFSA membranes' dual barrier functionality against both hydrogen and vanadium ions; therefore, we further investigated their in situ cell performance and stability. A schematic diagram of the PEMWE cell configuration and working mechanism is first provided in Figure 3a, where oxygen is generated at the anode and hydrogen is produced at the cathode. Polarization tests of the cell reveal better performance for the MXene‐coated PFSA membranes versus the pristine PFSA membrane at 60°C and 80°C (Figure S13). The polarization curves show near‐overlapping behavior at current densities below 1 A cm^−2^ (or voltages below 1.6 V). Beyond 2 A cm^−2^, the performance disparity among the curves becomes increasingly pronounced. Elevated temperature from 60°C to 80°C boosts current density at fixed voltage, owing to enhanced catalyst activity and ionomer conductivity that reduce activation and ohmic losses, consequently improving reaction kinetics and mass transport. In PEMWE, the PFSA‐1.5 M membrane reaches an optimal current density, achieving 5.4 A cm^−2^ at 2.0 V (60°C) and outperforming that of the PFSA membrane (4.6 A cm^−2^) under identical conditions (Figure 3b). When the temperature is elevated to 80°C, the current density of the cell with PFSA‐1.5 M membrane further escalates to 6.8 A cm^−2^ at 2.0 V, marking a 21.4% enhancement relative to that with the pristine PFSA membrane (5.6 A cm^−2^) (Figure 3c).

**FIGURE 3:**
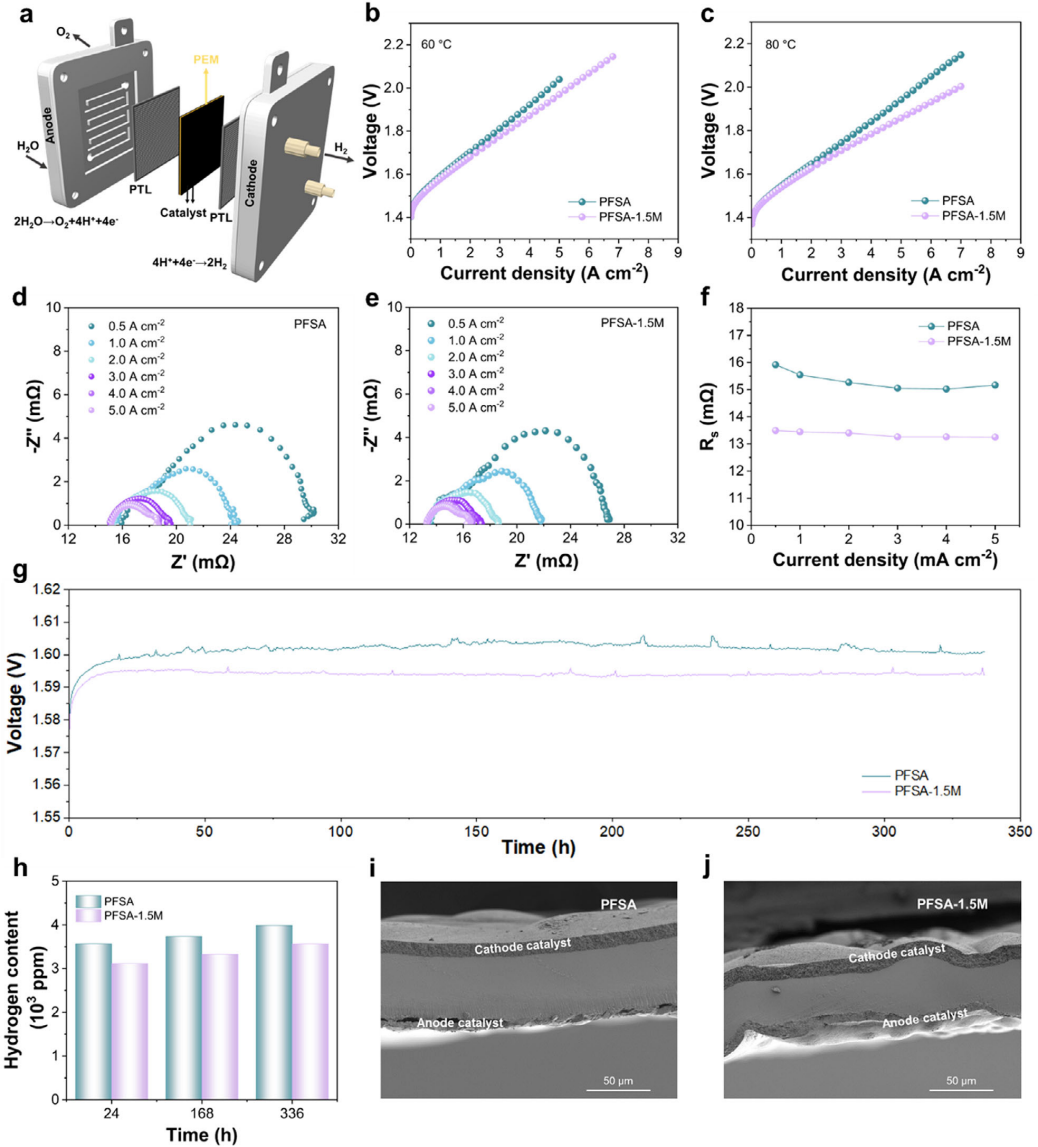
(a) Schematic illustration of the PEM water electrolyzer and its working principle; polarization curves of cell with PFSA membrane and PFSA‐1.5 M membrane at (b) 60°C and (c) 80°C; Nyquist plots from in situ EIS measurements under varying current densities at 60°C for (d) PFSA membrane and (e) PFSA‐1.5 M membranes; (f) fitted R_s_ values extracted from the Nyquist spectra; (g) durability curves of PFSA and PFSA‐1.5 M membranes under 1 A cm^−2^ at 60°C; (h) hydrogen content of anode outlet during durability test; cross‐sectional SEM images of (i) PFSA and (j) PFSA‐1.5 M membrane after PEMWE durability test.

Electrochemical Impedance Spectroscopy (EIS) analysis was used to help understand relevant aspects such as membrane, catalyst, and mass transport limitations to the cell polarization. Figure 3d,e present the Nyquist plots derived from EIS measurements of PEMWE systems utilizing PFSA and PFSA‐1.5 M membranes at 60°C under varying current densities. An equivalent circuit model, depicted in Figure S14, was employed to elucidate the contributions of ohmic resistance (*R_s_* ​), cathode and anode charge transfer resistances (*R_ct,c_​* and *R_ct,a​_*), and mass transfer resistance (*R_m_* ​) to the total impedance of the PEMWE system [57, 58]. This analysis facilitates a detailed decomposition of the electrochemical behavior under operational conditions. The high‐frequency intercept on the x‐axis of the Nyquist plots corresponds to the ohmic resistance (*R_s_* ​), primarily attributed to the electronic resistance of the cell components, with the membrane resistance being the dominant contributor [59]. As depicted in Figure 3f, the lower *R_s_* ​ values of the PFSA‐1.5 M membrane across varying current densities indicate its reduced resistance compared to the PFSA membrane. This observation aligns with the enhanced proton conductivity of the PFSA‐1.5 M membrane, as demonstrated in Figure 2f.

The small arc at high frequency corresponds to the cathode charge transfer resistance (*R_ct,c_* ​), while the large arc at intermediate frequencies is attributed to the anode charge transfer resistance (*R_ct,a_​*) [57, 58]. This distinction arises because the anode reaction kinetics are slower due to the requirement of four electrons for oxygen production [60]. Both *R_ct,c_* ​ and *R_ct,a_* decrease with increasing current density for PFSA membrane and PFSA‐1.5 M membrane (Figure S15), reflecting enhanced reaction kinetics. At current densities above 2 A cm^−2^, *R_ct,c_* ​ becomes particularly challenging to quantify precisely, as it is associated with the hydrogen evolution reaction (HER) kinetics, characterized by low resistance. Additionally, an *R_m_* arc appears in the low‐frequency range, becoming obvious in both membrane configurations (Figure 3d,e). The low‐potential regime (1.5 V) is dominated by activation polarization, while the high‐potential regime (2.0 V) is governed by ohmic or concentration polarization [58]. At current densities exceeding 2 A cm^−2^, *R_m_* becomes notable and increases with rising current density (Figure S15). This phenomenon may be attributed to enhanced bubble accumulation near the anode catalyst layer outlet, which hinders liquid water accessibility to active sites for electrochemical reactions [57]. Overall, *R_s_​* contributes the largest proportion to the total impedance, followed by *R_ct,a_*. The cell equipped with PFSA‐1.5 M membrane achieves lower *R_s_​* and *R_ct,a_* ​, contributing to its enhanced performance in PEMWE systems. Post‐durability EIS analysis reveals that although all resistance components (*R_s_*, *R_ct_*, and *R_m_*) increased for both membranes (Figures S16 and S17), the PFSA‐1.5 M membrane maintains lower values than pristine PFSA, indicating better stability under PEMWE operation.

The durability of the membranes was evaluated by monitoring the voltage evolution at 60°C under 1 A cm^−2^. Figure 3g shows that the PEMWE cell with PFSA‐1.5 M membrane maintains a lower cell voltage (∼1.594 V) than the PFSA membrane over 350 h, with stable performance after the initial 24 h. This confirms the PFSA‐1.5 M membrane's superior performance and durability during water electrolysis. Gas chromatography analysis indicates the PFSA‐1.5M‐based system produces ∼12% less hydrogen in the anodic oxygen stream during durability testing (Figure 3h), underscoring its improved hydrogen barrier capability. Post‐test SEM cross‐sectional morphology of the membranes reveals that the MXene‐containing barrier layer exhibits excellent integration with the PFSA matrix without delamination or cracking (Figure 3i,j). The minor separation between the catalyst layer and the membrane stems from the differential swelling behavior of the membrane and the catalyst during testing. The Ti concentration from anode outlet measured by ICP‐MS was extremely low (in the ppb range, as detailed in Figure S18), confirming that MXene decomposition and shedding from the membrane coating are minimal. Collectively, these durability metrics conclusively validate the exceptional performance and long‐term stability of the PFSA‐1.5 M membrane in PEMWE applications.

### 2.4 VRFB Performance and Durability

Figure 4a schematically illustrates a conventional VRFB configuration and working mechanism, where electrolytes stored in tanks are cycled through the stack during charge‐discharge processes. The performance of VRFB single cells equipped with PFSA and PFSA‐1.5 M membranes was evaluated by analyzing CE, voltage efficiency (VE), and EE under different current densities (80, 110, 160, 200 mA cm^−2^), as illustrated in Figure 4b. CE monotonically increases with increasing current density, whereas VE follows an inverse trend. These efficiency shifts are attributed to accelerated charge‐discharge kinetics dominating at higher current densities, alongside amplified ohmic polarization caused by elevated ionic transport resistance [36]. The PFSA‐1.5 M membrane demonstrates superior CE (95.0%−96.3%) over pristine PFSA (94.3%−95.7%) across all current densities, owing to its improved vanadium ion barrier capability through MXene‐mediated interfacial interactions and lamellar structural confinement. This confirms the efficacy of MXene‐PFSA synergy in suppressing vanadium ion crossover during VRFB operation. VE, governed by ohmic resistance and proton conductivity [8, 61], shows a slight enhancement in PFSA‐1.5 M membrane due to its marginally improved proton transport. But the increment is moderated by competitive cation migration between protons and charged vanadium species. Consequently, the PFSA‐1.5 M membrane achieves higher EE than PFSA, as the EE is determined by the multiplication of CE and VE to reflect overall energy losses in cycling processes.

**FIGURE 4:**
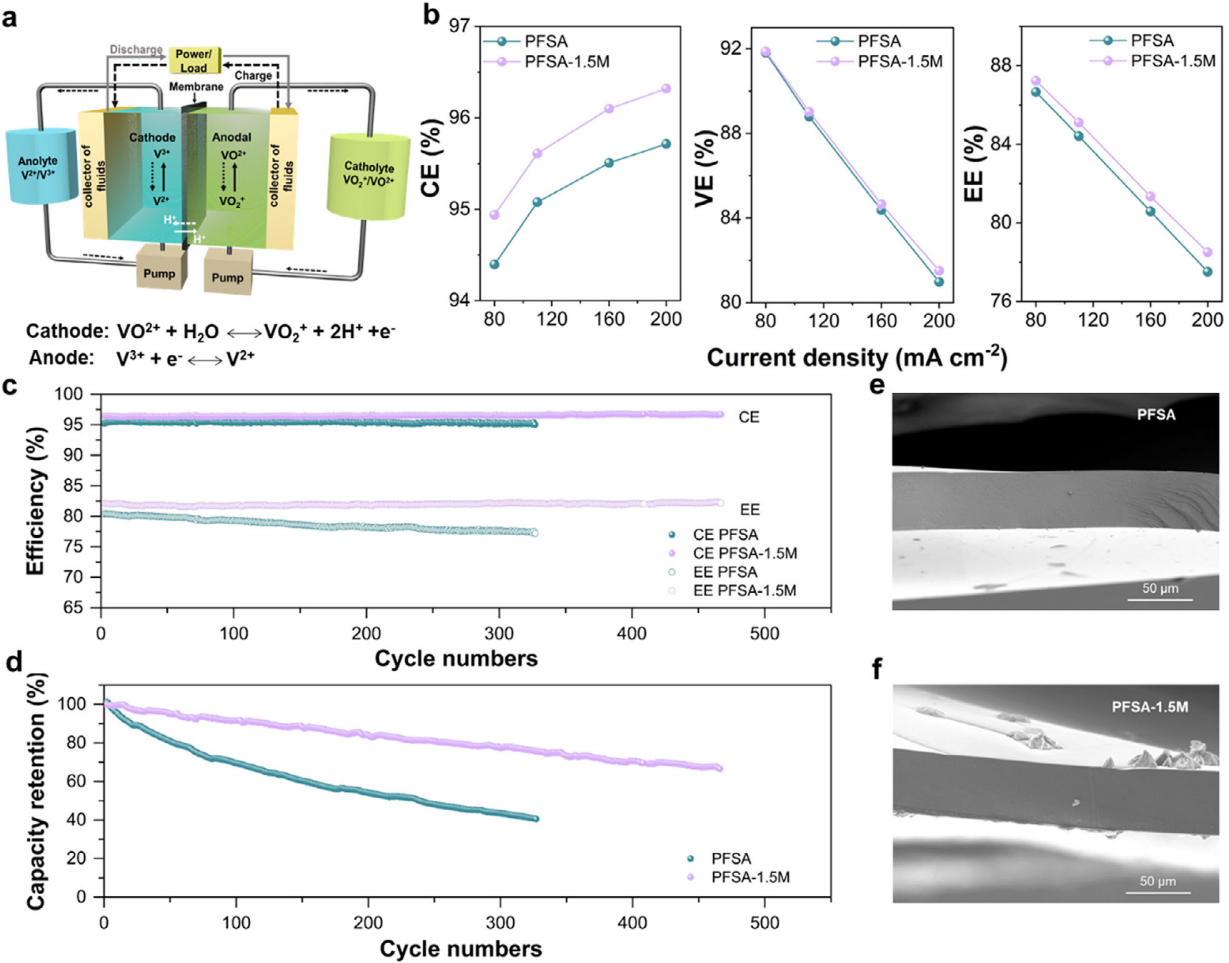
(a) Schematic of a VRFB configuration and working principle; (b) CE, VE and EE of the PFSA and PFSA‐1.5 M membranes; (c) CE, VE, and (d) capacity retention of PFSA and PFSA‐1.5 M membranes during multiple cycles. Cross‐sectional SEM images of (e) PFSA and (f) PFSA‐1.5 M membrane after VRFB durability test.

The operational durability of VRFB cells employing PFSA and PFSA‐1.5 M membranes was assessed via charge‐discharge cycles at 160 mA cm^−2^ without electrolyte remixing (Figure 4c). Compared with a decline for the PFSA membrane, PFSA‐1.5 M maintains stable CE (≈97%) during cycling, resulting from its MXene‐enhanced barrier effect against vanadium crossover. Furthermore, the PFSA‐1.5 M membrane achieves higher and more stable EE within ∼500 cycles compared to PFSA, which even suffers a 3.2% EE reduction within 325 cycles. The mitigated efficiency degradation verifies the PFSA‐1.5 M membrane's superior cycling durability in practical VRFB operation.

Figure 4d compares the discharge capacity retention between PFSA and PFSA‐1.5 M membranes. PFSA‐1.5 M exhibits superior stability, retaining 67% capacity after 470 cycles, versus PFSA's 40% retention after 325 cycles (terminated due to membrane failure), representing a 67.5% improvement. This disparity originates from the PFSA‐1.5 M membrane's enhanced ion selectivity, which effectively mitigates vanadium crossover. Post‐durability SEM cross‐sectional images show the MXene barrier layer remains intact within the PFSA matrix without delamination (Figure 4e,f). Moreover, the dry‐wet cyclic aging tests on the PFSA‐1.5 M membrane was conducted with result of no visible delamination, cracking, or detachment at the interface between the coating layers and the PFSA substrate (Figure S19). Both of these confirmed the stable interfacial adhesion between barrier layer and PFSA matrix. Although methods of electrolyte rebalancing or component redesign address vanadium ion crossover issues, membranes with high ionic selectivity remain one of the most promising strategies. The PFSA‐1.5 M membrane's dual merits—robust crossover resistance and proton conductivity—position it as a competitive candidate for durable VRFB application.

To further verify the chemical stability of MXene under operating conditions, XPS analysis was conducted on the membranes after durability testing in both PEMWE and VRFB systems. As the catalyst layer could not be separated from the membrane without damaging the underlying structure, the PFSA‐1.5 M catalyst‐coated membrane (CCM) after PEMWE durability testing was directly employed for XPS analysis. The full XPS spectra of all samples appear similar, with strong C, O, and F signals originating from the PFSA matrix, and no obvious Ti peaks can be identified (Figure S20a). The high‐resolution C 1s, O 1s, and F 1s spectra (Figure S20b–d) of the three tested samples (PFSA‐1.5 M before test, after VRFB, and PEMWE durability test) are largely similar to that of pristine PFSA. However, we observed slight shifts of certain peaks toward lower binding energies, which are most likely composite signals arising from structural evolution of the composite membrane under the harsh electrochemical environments. The Ti 2p high‐resolution XPS spectra (Figure S21) show that no Ti signals are observed for the pristine PFSA membrane. For the PFSA‐1.5 M membrane prior to testing, two distinct peaks are detected at ∼ 459 eV and ∼ 453 eV, which correspond to Ti═O and Ti─C, respectively, consistent with the pristine MXene. If substantial oxidation of MXene to TiO_2_ had occurred, a significant increase in the Ti═O peak intensity (associated with TiO_2_) and a concurrent decrease in the Ti─C peak intensity would be anticipated. However, after durability testing in both VRFB and PEMWE systems, the peak positions and the relative intensity ratios of Ti═O to Ti─C remain essentially unchanged compared with the pristine composite membrane. These results confirm that no significant degradation or oxidation of MXene took place under the tested operating conditions.

In the interfacial layer, the MXene nanosheets are embedded within the PFSA polymer matrix rather than being exposed as a bare layer on the membrane surface. This polymer encapsulation provides a physical barrier that limits direct contact between MXene and the oxidative species, thereby significantly slowing down the oxidation kinetics. It has been well documented that MXene oxidation is fastest in liquid media and slowest in solid media, including polymer matrices [62]. By dispersing MXene within the PFSA matrix, we effectively create a solid‐state encapsulation environment that protects the nanosheets from oxidative degradation.

Furthermore, the PFSA matrix itself contains abundant ─SO_3_H groups that can form hydrogen‐bonding interactions with MXene surface terminations (─OH, ─O, ─F). These interfacial interactions not only improve the dispersion and anchoring of MXene nanosheets within the coating layer but also provide additional chemical stabilization by passivating the reactive edge sites that are most susceptible to nucleophilic attack by water molecules [63]. The sandwich configuration ensures that the MXene‐containing layers are physically protected from direct exposure to the highly oxidative environment, while the two outer PFSA/MXene layers provide the functional benefits of enhanced proton conductivity and size‐exclusion selectivity. This design effectively combines the functional advantages of MXene with the protective role of the polymer matrix, addressing both performance and stability requirements.

### 2.5 Density Functional Theory (DFT) Simulations and Nanostructure Analysis by Waxs and Saxs

To further validate the effective composition of MXene‐involving coating layer, we used density functional theory (DFT) as a qualitative mechanistic probe to isolate the intrinsic interactions between species (Hydrogen, vanadium, and proton) and MXene/PFSA system. (Figure 5a–i). Detailed hydrated interface simulations, which would involve considerable complexity due to diverse hydration configurations and dynamic hydrogen bonds, are reserved for future work. The results reveal that hydrogen diffusion energy barriers across standalone lamellar MXene layers exhibit a slight initial increase followed by a decrease, remaining at relatively low levels overall. In contrast, blending MXene with PFSA induces interfacial interactions between sulfonic acid groups of PFSA and functional groups of MXene. This interaction significantly elevates hydrogen diffusion energy barriers within the MXene/PFSA coating layer, particularly when hydrogen traverses interfacial regions between sulfonic acid groups and MXene surface functional groups (B' site) (Figure 5a,b). The energy barrier of intermediate transition states increases from 0.19 eV of MXene to 0.51 eV of the MXene/PFSA system (B to B' site, Figure 5c), indicating greater difficulty in hydrogen permeation in the MXene/PFSA system. For vanadium ion (VO^2+^) crossover, similarly, the energy barrier for transport within the pristine MXene coating layer is lower than that within the MXene/PFSA composite coating layer (Figure 5d–f). The highest energy barrier occurs when vanadium ions pass through the interfacial region where sulfonate groups and MXene functional groups interact (E' site). The energy barrier of intermediate transition states increases from 0.18 eV of MXene to 0.65 eV of the MXene/PFSA system (E to E' site, Figure 5f). This region imposes a significant steric hindrance effect on vanadium ion permeation. Besides the size sieving effect, a Donnan effect further impedes vanadium ion transport due to the negative charge of the sulfonate groups. However, the results show that the calculated proton diffusion barrier (Figure 5g,i) is lower than those of hydrogen molecules and VO^2+^. More importantly, when the proton diffuses through the region where MXene interacts with the sulfonic acid groups of PFSA (H’ sites), the diffusion barrier further decreases (from 0.22 to 0.15 eV). This reduction suggests that additional hydrogen bonds are formed between the functional groups of MXene and the sulfonic acid groups, providing new and more favorable hopping sites for proton transport. Consequently, the synergistic integration of MXene and PFSA within the composite coating layer achieves exceptional hydrogen and vanadium ion barrier efficacy. The combined DFT results clearly establish the selectivity of the PFSA/MXene composite membrane: protons are preferentially transported, while the crossover of hydrogen and vanadium ions is effectively suppressed.

**FIGURE 5:**
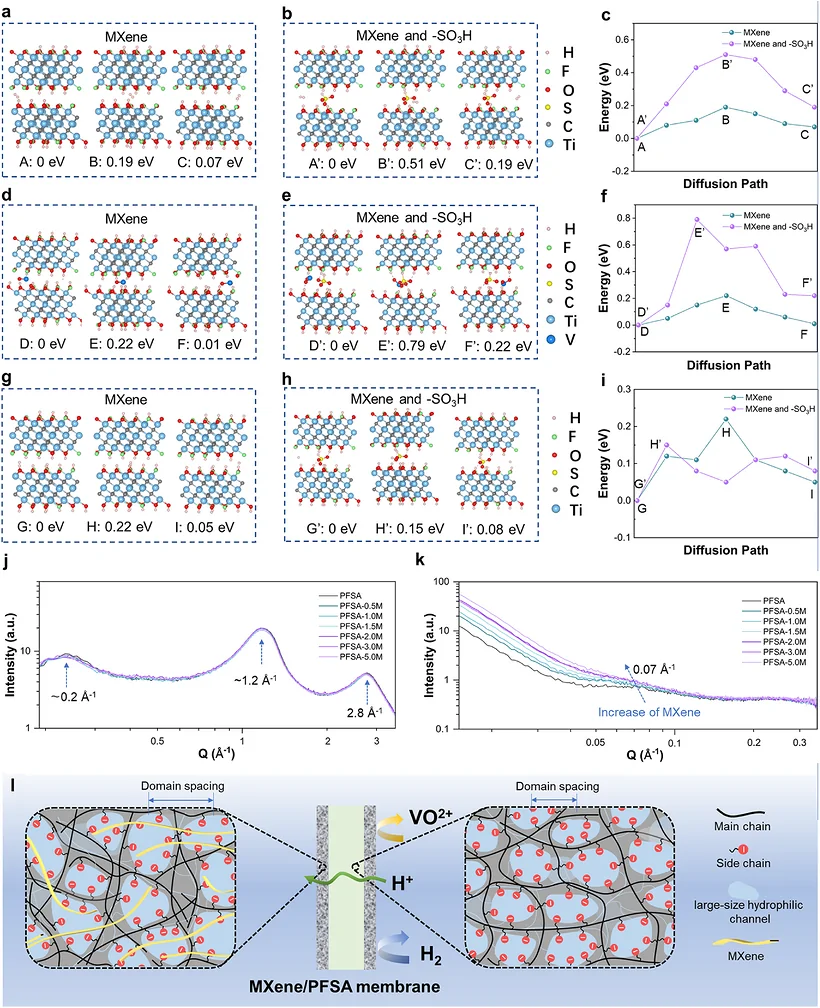
DFT simulations of hydrogen diffusion pathway in (a) MXene and (b) MXene/ −SO_3_H system; (c) hydrogen diffusion energy; vanadium ion crossover pathway in (d) MXene and (e) MXene/ −SO_3_H system; (f) vanadium ion diffusion energy; proton transport pathway in (g) MXene and (h) MXene/ −SO_3_H system; (i) proton diffusion energy; (j) WAXS profiles and (k) SAXS profiles of PFSA and MXene/ PFSA membranes. (l) schematic illustration of the hydrogen/ ion barrier mechanism for the MXene/PFSA membrane.

To investigate the influence of MXene in the barrier layer on the structure of the PFSA matrix and the underlying barrier mechanism, Wide‐Angle X‐ray Scattering (WAXS) and Small‐Angle X‐ray Scattering (SAXS) analyses were conducted for the membranes. The structural differences between the composite and pristine PFSA membrane primarily originate from the MXene‐containing coating layer. The WAXS profiles (Figure 5j) reveal three characteristic peaks. The fitted and calculated domain spacing (Bragg's Law) and crystal size (coherence lengths, by Scherrer formula) are listed in Table S3. The scattering features at ∼1.17 Å^−1^ and 2.8 Å^−1^ are associated with the ordered structure of the PTFE backbone. The 1.17 Å^−1^ peak reflects an interchain spacing of 5.4 Å (crystal size of 30.5 Å), matching the ordered arrangement of polymer segments (closely resembling that of PTFE) [12, 64]. The 2.8 Å^−1^ peak corresponds to a spacing of 2.2 Å (crystal size of 16.5 Å), representing the distance between CF_2_ groups in the PTFE backbone [53]. These two peaks remain nearly identical for both the composite and pristine membranes, indicating that MXene incorporation does not disrupt the ordered structure of the PTFE backbone. Additionally, a peak at ∼0.2 Å^−1^ in the low‐Q region is attributed to small‐sized ordered hydrophilic ionic clusters. The domain spacing is slightly increase from 26.2 Å of PFSA to 26.8 Å of PFSA‐5.0 M, indicating the MXene slightly expands the smaller size hydrophilic domains.

Relative to the peak at ∼0.2 Å^−1^, the peak at ∼0.07 Å^−1^ in the SAXS profile exhibits more pronounced changes (Figure 5k). This scattering peak corresponds to larger‐scale ordered structures with a spacing of ∼89.7 Å, representing the domain spacing of hydrophilic chain assemblies. Upon MXene incorporation, this peak shifts toward the lower Q region with a broader full width at half maximum (FWHM). When the MXene content exceeds 1.5 wt.%, the peak becomes indistinct, suggesting that the ordered structure tends to be disordered. These modifications imply that higher MXene loading expands the inter‐domain spacing and disrupts short‐range ordering of chain assemblies.

MXene primarily induces a steric effect on the large‐sized hydrophilic domains while exerting minimal influence on small‐sized ones (∼0.2 Å^−1^). The enlarged but disordered channels formed by chain assemblies could couple with reduced vanadium ion and hydrogen permeation, highlighting the physical blocking effect of MXene's 2D nanosheets. The relatively larger‐sized MXene is connected with multiple hydrophilic domains, and the functional groups interact with the −SO_3_^−^. The orderliness of large‐sized channels decreases with the increase of MXene content, which may increase the tortuosity of the channels and hinder the diffusion of larger vanadium ions and hydrogen molecules, thereby reducing the permeability of both species. However, proton transport, relying primarily on the Grotthuss (hopping) mechanism between hydrogen‐bonding network, is maintained or even enhanced due to the hydrogen‐bonding sites provided by MXene. As illustrated in Figure 5l, micron‐sized MXene sheets interconnected multiple hydrophilic channels, hindering vanadium ion crossover and hydrogen diffusion due to MXene's impermeability and the increased tortuosity after adding PFSA matrix. Simultaneously, the hydrogen‐bonding network formed between MXene's functional groups and SO_3_^−^ facilitates proton conduction.

In summary, both PEMWE and VRFB applications require high proton conductivity and low crossover of active species. However, distinct operating conditions impose different priorities: PEMWE operates at high anodic potentials with oxygen evolution, emphasizing oxidative stability and low gas crossover, while VRFB prioritizes ionic selectivity (proton/vanadium) over absolute conductivity due to continuous exposure to VO^2+^ species. Additionally, PEMWE favors thinner membranes to minimize ohmic losses, whereas VRFB benefits from thicker structures to suppress vanadium crossover. These commonalities and differences collectively justify the PFSA/MXene composite membrane as a promising candidate for both applications.

## 3 Conclusion

In summary, we have demonstrated that MXene‐regulated nanointerfaces can be used to decouple proton conductivity from molecular and ionic crossover in PFSA‐based membranes. By integrating MXene in the coating layers of PFSA, the composite membranes achieve higher water uptake and reduced swelling properties. Meanwhile, it establishes a hydrogen‐bonding network between functional groups of MXene and sulfonate moieties of PFSA matrix, resulting in an optimal proton conductivity of 215.9 mS cm^−1^ at 1.5 wt.% MXene loading. Moreover, the PFSA‐1.5 M membrane achieves exceptional H^+^/H_2_ and H^+^/VO^2+^selectivity, 65.6% and 57.4% higher than the PFSA membrane, respectively. DFT simulations demonstrate higher diffusion energy barriers for hydrogen and vanadium ions and lower proton transport energy within the MXene/PFSA barrier layer, which is beneficial for inhibiting the permeation of both hydrogen and vanadium ions. Further validated by WAXS and SAXS technologies, MXene addition enlarges domain spacing and restructures to large‐sized hydrophilic channels, disordering the hydrogen/ion pathways. In PEMWE operation, the PFSA‐1.5 M membrane shows a superior current density of 6.8 A cm^−2^ at 2 V (21.4% higher than the pristine PFSA membrane) and maintains lower voltage (∼1.594 V) with reduced hydrogen content in oxygen (less than 3600 ppm) during durability testing at 60°C (1 A cm^−2^). For VRFB, it displays higher CE and EE, retaining 67% capacity after 470 cycles, whereas pristine PFSA degrades to 40% after 325 cycles. This work underscores the broader potential of 2D MXene as interfacial regulators for ion‐selective transport in polymer membranes, providing new design principles for next‐generation electrochemical energy technologies.

## Author Contributions

The experiments were conducted and the manuscript was written by M.J. and P.G., with the original idea conceived by P.G. Y.D. and D.Y. oversaw the project, providing research facilities and funding. Valuable insights on characterization and manuscript improvements were offered by D.W., H.L., T.L. and Z.S. Assistance with characterization and DFT calculations was provided by Y.J., Y.Z., W.L., L.Z. and K.L.

## Conflicts of Interest

The authors declare no conflicts of interest.

## Supporting information




**Supporting File**: advs78270‐sup‐0001‐SuppMat.docx.

## Data Availability

The data that support the findings of this study are available from the corresponding author upon reasonable request.
