# Supplementary material for: Dual‐Functional Membranes With Interfacial MXene/PFSA Nanoarchitectures for Low Hydrogen Permeation in Water Electrolysis and Reduced Vanadium Ion Crossover in Flow Batteries
Source: Adv Sci (Weinh). 2026 Oct 14:e78270. Online ahead of print. doi: 10.1002/advs.78270 (PMC13668059; doi:10.1002/advs.78270)
Supplement: Supplementary file 1 [file ADVS-9999-e78270-s001.docx]

**Dual**-**Functional Membranes with Interfacial MXene/PFSA Nanoarchitectures for Low Hydrogen Permeation in Water Electrolysis and Reduced Vanadium Ion Crossover in Flow Batteries**

Panpan Guan^1^, Mingjia Jiang^1^, Yunhu Jiang^2^, Wei Li^1^, Yecheng Zou^2^, Lixiao Zhang^1^, Kaifeng Long^1^, Ziqi Sun^3^, Ting Liao^4^, Huakun Liu^1^, Ding Yuan^1^, Dingsheng Wang^5^, Shi Xue Dou^1^, and Yuhai Dou^1^*

^1^ Institute of Energy Materials Science (IEMS), University of Shanghai for Science and Technology, Shanghai 200093, China

^2^ Shandong Key Laboratory of Core Materials and Technologies for Hydrogen Energy and Dongyue Future Hydrogen Energy Materials Company, Zibo 256401, China

^3^ School of Chemistry and Physics, Queensland University of Technology, Brisbane 4000, Australia

^4^ Faculty of Engineering, School of Mech., Medical & Process Engineering, Queensland University of Technology, Brisbane 4000, Australia

^5^ Engineering Research Center of Advanced Rare Earth Materials, Department of Chemistry, Tsinghua University, Beijing 100084, China

Corresponding email: [y.dou@usst.edu.cn](mailto:y.dou@usst.edu.cn)

# Experimental Section

**Materials:**

Ti_3_AlC_2_ precursor powder (200 mesh) was purchased from 11 Technology Co., Ltd. (Jilin, China). Long side-chain PFSA polymer (IEC: 1.1 mmol g^-1^), supplied by Dongyue Shenzhou New Materials (Zibo, China), was used as received. Isopropanol and ethanol were obtained from Sigma-Aldrich (St. Louis, MO, USA) and used without purification. Hydrochloric acid (HCl) and lithium fluoride (LiF) were sourced from Sinopharm Chemical Reagent (Shanghai, China) and Energy Chemical (Shanghai, China), respectively. Deionized water was used in all experiments.

**Preparation of MXene:**

Initially, 2.4 g of LiF was dissolved in 30 mL of 9 M HCl under vigorous stirring at room temperature to form a homogeneous solution. Subsequently, 1.5 g of Ti_3_AlC_2_ powder was incrementally added into the acidic mixture, followed by continuous stirring at 35 °C for 48 h to ensure complete etching of the Al layer. The reacted slurry was washed with 1 M HCl and then deionized (DI) water with 3500 rpm centrifugation until the supernatant reached a pH of ~6, effectively removing residual ions and byproducts. The purified sediment was redispersed in DI water via 30 min of ultrasonication to delaminate stacked layers. After centrifugation at 3500 rpm for 50 min, the upper colloidal suspension enriched with multilayer Ti_3_C_2_T_x_ was carefully decanted. To achieve a few-layer MXene, the collected multilayer dispersion underwent further exfoliation through high-power ultrasonication (250 W, 1 h) at 5 °C, and the supernatant was collected. The concentration of the supernatant containing few-layer Ti_3_C_2_T_x_ was calculated to be 24.9 mg/ml by vacuum filtration through a PTFE membrane with a pore size of 0.22 μm.

**Membrane preparation:**

The PFSA polymer was dissolved in isopropanol/water/ethanol (50/45/5 wt%) mixed solvent to form a 15 wt% dispersion. A certain amount of the prepared fewer-layer Ti_3_C_2_T_x_ supernatant was added to the 15 wt% PFSA dispersion, stirred at 500 rmp at RT for 30 min, and ultrasonicated in a 5 ℃ bath (200 W)for 2 h to form the Ti_3_C_2_T_x_/PFSA homogeneous dispersion. The content of MXene to the PFSA was 0.5 wt%, 1.0 wt%, 1.5 wt%, 2.0 wt%, 3 wt%, and 5 wt%.

The PFSA membranes were fabricated by casting the PFSA dispersion on a glass plate using a doctor blade, dried at 80 °C for 30 min, and annealed at 145 °C for 45 min. After the annealing process, the annealed membranes were quenched to room temperature and peeled off from the glass substrate.

The MXene/PFSA composite membrane was prepared as follows: a MXene/PFSA dispersion was blade-coated onto a glass substrate and dried in a vacuum oven at 85 °C for 20 min to form the initial layer. Secondly, a PFSA dispersion layer was similarly cast and dried on top of the preceding MXene/PFSA layer using the same drying process to form the middle layer. A final MXene/PFSA dispersion was coated onto the bilayer structure, followed by vacuum drying at 85−90 °C for 15−20 min to complete the multilayer assembly. The obtained three-layer membrane was transferred to an air-drying oven preheated to 85 °C and annealed at 145 °C for 45 min to enhance structural integrity and interfacial bonding. The resultant MXene/PFSA-coated membranes were systematically designated as PFSA-xM, where “x” denotes the MXene mass percentage relative to the PFSA matrix in the coating layers. For instance, the PFSA-1.5M membrane indicates the coating layer containing 1.5 wt% MXene within the PFSA matrix.

**Characterization:**

X-ray photoelectron spectroscopy (XPS, AXIS SUPRA+, Shimadzu Company, Japan) was used to investigate the surface chemical compositions of Ti_3_C_2_T_x_ filler. X-ray diffraction (XRD) patterns were collected by an X-ray diffractometer (SmartLab, Rigaku, Japan) equipped with Cu Kα radiation. The scanning speed is 5° min^-1^ in the 2θ range of 5−80°. The interplanar spacing is calculated using the Bragg equation, nλ = 2dsinθ, where n = 1, if not specified, λ represents X-ray wavelength (𝜆 of Cu Kα radiation ≈0.15406 nm), and θ represents the diffraction angle. The membrane thermogravimetric loss was obtained by TG (TG 209 F1 Libra, NETZSCH, Germany). Gas chromatography (GC-2014, SHIMADZU, Japan) was used to determine the content of hydrogen in oxygen. The ultraviolet spectrometer (UV, UV-3600i Plus, SHIMADZU, Japan) was used to detect the content of vanadium ions. The contact angle test (ZJ-6900, Z. Jia equipment, China) was adopted to evaluate the wettability of water on the membrane surface. A field-emission scanning electron microscope (SEM, Thermo Apreo 2S HiVac, Thermo Fisher Scientific, USA) was used to observe the morphology of the raw material Ti_3_AlC_2_, the prepared Ti_3_C_2_T_x_ filler, and the membranes. Small-Angle X-ray Scattering (SAXS) and Wide-Angle X-ray Scattering (WAXS) of the membranes were performed using Xeuss 3.0, Xenocs China, China. The experiments were carried out at room temperature with X-ray radiation of a wavelength of λ = 1.35 Å (i.e., with energy of 9.18 keV). The sample to charge-coupled device (CCD) distance is ~782 mm for SAXS and ~ 54.7 mm for WAXS, and the data were analyzed with the Igor Pro software. The mechanical properties of the samples were tested using an electromechanical universal testing machine (UTM4102HB, Shenzhen Suns Technology Stock Co., LTD, Shenzhen, China) equipped with a 100 N load cell (temperature: 25 ± 1 °C; relative humidity: 50 ± 5%). The rectangular samples of 1 cm (width) × ~2 cm (length) were stretched at a speed of 50 mm/min. The membranes were dried in an oven at 80 ℃ overnight before testing. At least five specimens for each membrane sample were tested and the average was calculated for data analysis. Low-field ^1^H NMR relaxometry was performed on a 12 MHz NMR spectrometer (MicroMR12-025V) using a Carr-Purcell-Meiboom-Gill (CPMG) pulse sequence. The echo time (TE) was set to 0.08 ms with 5000 echoes collected, and the recycle delay (TW) was 2500 ms. The 90° pulse width (P1) was 6.8 μs, and 32 scans were accumulated for each measurement. The transverse relaxation time (T_2_) was extracted by fitting the CPMG decay curves with a multi-exponential function. The solid-state ¹H NMR (Bruker Avance NEO 400WB) were recorded on a Bruker Avance 400 MHz spectrometer. Chemical shifts were externally referenced to tetramethylsilane (TMS). ICP-MS (Thermo Fisher Scientific iCAP RQ) was used to test the Ti content of anode outlet during PEMWE test.

**Water uptake and swelling ratio:**

Membrane samples for water swelling were cut into 2.5 cm × 4.5 cm rectangles. Water uptake was calculated from the weights of “dry” and “wet” membranes. The “dry” state was achieved by dehydrating membranes under vacuum at 90 °C for 12 h. The “wet” state involved hydrating membranes in DI water for one hour at the desired temperatures. Weights of wet membranes were measured within one minute after removal from water, using filter paper to blot excess surface water.

Water uptake is calculated by equation (1)

Water uptake (%) = $\frac{\text{W}_{\text{wet}}\text{-}\text{W}_{\text{dry}}}{\text{W}_{\text{dry}}}$ $\text{×100\%}$	(1)

Where $W_{wet}$ and $W_{dry}$ are the weights of “wet” and “dry” membranes.

Swelling ratio (%) was calculated by equation (2):

Swelling ratio (%) **=** $\frac{\text{L}_{\text{wet}}\text{-}\text{L}_{\text{dry}}}{\text{L}_{\text{dry}}}$ $\text{×100\%}$	(2)

Where L_dry_ and L_wet_ are the lengths of the “dry” and “wet” membranes, respectively, cm.

**Proton conductivity:**

Proton conductivity for the membranes in water was measured using a two-probe conductivity cell connected to an electrochemical test system (Autolab PGSTAT 302N, Metrohm, Switzerland), with the frequency range from 1 to 1 MHz and an oscillating amplitude of 10 mV. The conductivity was calculated from the semi-cycle resistance of the Nyquist plot obtained by AC impedance spectroscopy. The membrane samples for proton conductivity at different temperatures were equilibrated for 30 min at the set temperatures. The calculation of proton conductivity (σ) was according to the following equation (3):

$\text{ }\text{σ}\text{ }\text{= }\frac{\text{L}}{\text{A×R}}$	(3)

where σ is the proton conductivity, mS cm^-1^. *L* is the distance between two electrodes, cm. *R* is the measured ohmic resistance, KΩ, and *A* is the cross-sectional area of the membrane sample, cm^2^.

The activation energy (E_a_) for each membrane was calculated using the Arrhenius equation, as reported in references ^[1,2]^:

σ = σ_0_ exp (−E_a_/RT)	(4)

σ and σ_0_ are the values of the conductivity and pre-exponential factor, respectively, S/cm, E_a_ is the activation energy required for protons to transport, KJ/mol, R is the gas constant, Jmol^−1^ K^−1^, and T is the absolute temperature, K. In general, activation energy less than 40 KJ mol^-1^ is required for proton hopping in the Grotthuss mechanism, while a higher energy (E_a_ > 40 KJ mol^-1^) is required to transport the larger ionic complexes in the vehicular mechanism. ^[3,4]^

**Ion-exchange capacity (IEC):**

The ion exchange capacities (IECs, mmol/g) of the membranes were determined by a method of back-titration. Dried (200-300mg) membranes were first equilibrated for 48 h in 0.2 M NaCl at room temperature with regular agitation to release the proton. The solutions were then back titrated with NaOH (0.01M) by a Metler Toledo T50 automatic titrator. The value of IEC was obtained from an average of three samples for one type of membrane. The IEC was calculated as equation (5):

IEC = $\frac{C_{NaOH}\times V_{NaOH}}{W_{dry}}$	(5)

Where $C_{NaOH}(mol/L)$, $V_{NaOH}$(mL), and Wdry (g) are the concentration and volume of the consumed NaOH, and the dry weight of the membrane sample, respectively.

**Hydrogen permeability:**

All membranes were cut into a rectangle with an area of 16 cm^2^ for gas permeation measurements. The permeability of H_2_ was determined by a pressure-difference method using a membrane permeability analyzer (VAC-V1, Labthink Instrument, China).

**Density Functional Theory (DFT):**

All the first-principles spin-polarized calculations were performed by using the Vienna ab initio Simulation Program (VASP).^[5, 6]^ The generalized gradient approximation (GGA) in the Perdew-Burke-Ernzerhof (PBE) form and a cutoff energy of 500 eV for the planewave basis set were adopted.^[7]^ A 2 × 2 × 1 Monkhorst-Pack^[8]^ k grid was used for sampling the Brillouin zones at the structure calculation. The ion-electron interactions were described by the projector augmented wave (PAW) method.^[9]^ The convergence criteria of structure optimization were chosen as the maximum force on each atom less than 0.02 eV/Ǻ with an energy change less than 1 × 10^-5^ eV. To calculate the kinetic energy barrier of diffusion, the climbing image nudged elastic band (CI-NEB) method was used to search for the transition states.^[10]^

**Vanadium crossover:**

Vanadium crossover, the diffusion of vanadium ions across the membrane, causes coulombic efficiency (CE) loss, capacity decay, and open-circuit voltage (OCV) decline during VRFB cycling. To determine the diffusion coefficients of penetrating VO^2+^ ions through selected membranes, we employed an H-type diffusion cell. The membrane, sandwiched between the two half-cells using rubber gaskets, has an exposed area of 0.785 cm^2^ in each electrolyte compartment. A total of 10 mL of 1 mol L^-1^ VOSO_4_ in 2 mol L^-1^ H_2_SO_4_ was added to the left half-cell and 10 mL of 1 mol L^-1^ MgSO_4_ in 2 mol L^-1^ H_2_SO_4_ to the right half-cell. Both solutions were magnetically stirred at 200 rpm to prevent concentration polarization, with MgSO_4_ equalizing ionic strength and minimizing osmotic pressure effects. VO^2+^ concentration in the right-side solution was measured by UV-Vis spectroscopy (at RT) at selected time intervals using 2 mL samples. Samples were immediately returned after UV measurement. A pre-experiment calibration curve for VO^2+^ solutions of different concentrations was established (**Figure S5**), for which the characteristic peak absorbance at 760 nm was used as a reference. VO^2+^ crossover through each membrane was compared, and diffusion coefficients were calculated using Equation (6):^[11]^

$\text{V}_{\text{2}}\text{∙}\frac{\text{d}\text{C}_{\text{2}}\left( \text{t} \right)}{\text{dt}}\text{= A∙}\frac{\text{D}}{\text{L}}\text{(}\text{C}_{\text{1}}\text{(t)-}\text{C}_{\text{2}}\text{(t))}$	(6)

where V_2_ is the permeate-side solution volume, mL. C_1_ and C_2_ are the VOSO_4_ concentrations on the feed side and permeate side, mol·mL⁻^1^, respectively. A is the membrane's effective area, cm^2^. D is the VO^2+^ diffusion coefficient, cm^2^·s⁻^1^. L is the membrane thickness, cm. t is testing time, s. Due to lower VO^2+^ concentration on the permeate side, enrichment-side (feed-side) concentration changes are negligible. Thus, Equation (6) simplifies to Equation (7):

$\text{C}_{\text{2}}\text{= A∙}\frac{\text{D∙C}_{\text{1}}\left( \text{0} \right)}{\text{V}_{\text{2}}\text{∙L}}\text{∙t}$	(7)

There is a linear relation between C_2_ and time, with the slope (k) equal to $\text{A}\text{∙}\frac{{\text{D}\text{∙}\text{C}}_{\text{1}}\left( \text{0} \right)}{\text{V}_{\text{2}}\text{∙}\text{L}}$. Therefore, the diffusion coefficient (D) can be calculated as Equation (8):

$\text{ }\text{D}\text{ }\text{= }\frac{\text{K∙}\text{V}_{\text{2}}\text{∙L}}{\text{A∙}\text{C}_{\text{1}}\text{(0)}}$	(8)

**PEMWE test:**

Membrane Electrode Assemblies (MEAs) Preparation: Commercial catalytic layer (Shengshui New Energy Technology, Co., Ltd. Tech, anode loading: 1.0 mg cm^-2^ IrO_x_, cathode loading: 0.4 mg cm^-2^ Pt/C) was transferred onto both sides of the prepared membranes via hot-pressing (0.7 MPa, 150 °C, 10 min). Post-transfer, the membrane was edge-sealed and subjected to secondary hot-pressing (0.7 MPa, 150 °C, 5 min) to ensure interfacial flatness and good airtightness, yielding an MEA with an active area of 2 × 2 cm^2^.

Polarization Curve: The fabricated MEA was integrated into a PEM electrolyzer cell. Before testing, the MEA underwent electrochemical activation at 1 A cm^-2^ until steady-state performance was achieved. Polarization curves were measured at 60 ℃ and 80 ℃ under different current densities. The deionized water flow was 80 mL min^-1^.

Electrochemical Impedance Spectroscopy (EIS): EIS measurements were conducted by using an electrochemical workstation (DH7002A-1, Donghua Analysis, China) with a power amplifier (DH7101A, Donghua Analysis, China). The DC power from the electrochemical workstation was loaded on the PEM electrolyzer with an AC perturbation (5% amplitude). Spectra were acquired at 60 °C across multiple current densities (0.5, 1, 2, 3, 4, and 5 A cm^-2^).

Long-Term Stability Evaluation: MEAs with PFSA and PFSA-1.5M membranes (active area: 5 × 5 cm^2^) were fabricated using identical catalyst/hot-pressing protocols as those used for polarization curve test. Durability tests were performed at 1 A cm^-2^ and 60 °C for 336 h. The cell voltage was recorded at 30-minute intervals, and the hydrogen content in the anode oxygen output was analyzed during the durability test.

**VRFB test:**

The VRFB single-cell test was conducted at 35 ℃ using a membrane (active area: 8 cm × 6 cm) sandwiched between carbon felts and graphite bipolar plates. Initially, both catholyte and anolyte consisted of 1.7 mol L^-1^ VO^2+^ and V^3+^, respectively, in 4.5 mol L^-1^ H_2_SO_4_ as the supporting electrolyte, with flow rates fixed at 50 mL min^-1^. The circulating electrolyte volumes for both the anolyte and catholyte were 80 mL each. The temperature was maintained constant throughout cycling via a water bath circulation system, and prior to each extended cycling test, both the anode and cathode compartments were purged with nitrogen for protection. Charge-discharge test was operated under different current densities within a voltage window of 1.0−1.55 V. For stability assessment of the membrane and the cell performance, a cyclic test was conducted. Charge-discharge cycling test of the cell was carried out at 160 mA cm^-2^ at room temperature by a Neware CT-4008-5020A-NFA. For dry-wet cyclic aging tests on the PFSA-1.5M membrane, the test conditions were as follows: Dry state: 80 °C, 0% RH, 3 min; Wet state: 80 °C, 100% RH, 3 min. The membrane was subjected to 375 cycles under these alternating conditions. CE, VE, and EE were calculated using the following equations (9-12):

CE (%) = $\frac{\text{Discharge capacity}}{\text{Charge capacity}}$ × 100%	(9)

EE (%) = $\frac{\text{Discharge }\text{energy}}{\text{Charge energy}}$ × 100%	(10)

VE (%) = $\frac{\text{EE}}{\text{CE}}$ × 100%	(11)

Capacity retention = $\frac{\text{C}_{\text{n}}}{\text{C}_{\text{0}}}$ × 100%	(12)

Where C_0_ is the initial discharge capacity, C_n_ is the discharge capacity after the 𝑛^th^ cycle.

**Table S1. H_2_ Barrier, Proton Selectivity and Cycling Stability of MXene-Based Composite Membranes for PEMFC/DMFC/PEMWE Applications.**

| Composite membrane | Key design strategy | H_2_ barrier property | Proton selectivity | Cycling / OCV stability | Application | Ref. |
| --- | --- | --- | --- | --- | --- | --- |
| CNF/SA/MXene (CSM-IV) | Mixed-dimensional assembly + Ionic cross-linking | N/A | N/A  (proton conductivity: 45.4 mS cm^-1^) | OCV decay: 0.4 mV h^-1^ (58h) | DMFC | 12. Adv. Funct. Mater, 2025. |
| SPEEK/f-MXene/Pt-TiO_2_-5 | Stacking regulation + Self-humidifying | N/A | N/A  (proton conductivity: 143 mS cm^-1^) | N/A | DMFC, PEMFC | 13.Nanomaterials, 2026. |
| 4% MXene-Cu_2_O/SPEEK | Inorganic filler reinforcement | N/A | N/A  (proton conductivity: 10.5 mS cm^-1^) | 8 h stable H_2_ production | PEMWE | 14. Catalysis Today,  2023. |
| MXene/SPI-2SMH | Interface bonding + Radical scavenging | N/A | N/A  (proton conductivity: 313.9 mS cm^-1^) | ~ 17 h OCV stability | PEMFC | 15. J. Mater. Sci.,  2026. |
| SSC PFSA / Ti_3_C_2_T_x_ (1.5 wt%) | 2D filler physical barrier + Ionic domain regulation | H_2_ permeation: 2.1×10^-3^ cm^3^·cm^-2^·min^-1^ | N/A:  (proton conductivity: 251 mS cm^-1^) | N/A | PEMFC  (HT) | 16. Materials,  2021. |
| r-PEM / MXene–CeO_2_ (0.1 mg·cm^-2^) | Self-assembly + In-situ CeO_2_ growth on MXene (coating) | H_2_ crossover: 2.6 mA·cm^-2^ | N/A:  (proton conductivity: 85 mS cm^-1^) | OCV decay: 0.53 mV h⁻¹ (150 h) | PEMFC | 17. ACS Sustainable Chem. Eng., 2022. |
| PFSA/MXene-1.5 | Interfacial regulation + bifunctionality | H_2_ permeability:  25 barrer (80 ℃) | 8.4 mS cm^-1^ barrer^-1^( proton conductivity: 215.8 mS cm^-1^, 80 ℃) | 350 h stable OCV voltage (1.594 V) | PEMWE | This work |

Notes for Table S1: OCV = open-circuit voltage; N/A = not available.

**Table S2. VO^2+^ Rejection, Proton Selectivity and Cycling Stability of MXene-Based Composite Membranes (VRFB Applications).**

| Composite membrane | Key design strategy | VO^2+^ rejection | Proton selectivity | Cycling stability | Application | Refs. |
| --- | --- | --- | --- | --- | --- | --- |
| SPEEK/MS-10 | In-situ COF growth on MXene + Size sieving | 2.9×10^-8^ cm^2^·min^-1^ | 1.6×10^6^ S·min·cm^-3^ | 200 cycles at 120 mA cm^-2^, capacity decay: ~52%; EE retention: ~ 78% | VRFB | 18. J. Power Sources, 2025 |
| SPEEK/MXene-1 | PDA/PEI surface coating MXene + Proton hopping sites | 5.8×10^-10^ cm^2^·min^-1^ | 7.1×10^7^ S·min·cm^-3^ | 200 cycles at 120 mA cm^-2^, capacity decay: 60%; EE retention: ~ 78% | VRFB | 19. J. Polymer Sci., 2025 |
| PFSA/CF_3_SO_3_-MXene | Organic acid etching for MXene + pH-responsive Donnan exclusion effect | ~1.8×10^-3^ cm^2^·min^-2^ | 11.7 S·min·cm^-3^ | 800 cycles at 150 mA·cm^-2^, EE retention 84.2% | VRFB | 20. Adv. Funct. Mater., 2025. |
| SCNC/MXene/PVDF-HFP | 1D/2D synergistic assembly + H-bond networks | 4.92×10^-9^ cm^2^·min^-1^ | 3.21×10^6^ S·min·cm^-3^ | 90 cycles at 100 mA·cm^-2^, capacity decay: ~29%; EE retention 84.4% | VRFB | 21. Nano Lett., 2025. |
| MXene/PFSA | Interfacial regulation + bifunctionality | 0.90×10^-8^ cm^2^ s^-1^(RT)  Or  5.4×10^-7^ cm^2^ min^-1^(RT) | 102.4×10^8^ mS·s·cm⁻³  Or  1.7×10^5^ S·min·cm⁻³ | 470 cycles at 160 mA·cm⁻², capacity decay: ~33%; EE retention 82% | VRFB | This work |

Notes for Table S2: a) VRFB = vanadium redox flow battery; VO²⁺ = vanadyl ion; PDA = polydopamine; PEI = polyethyleneimine; COF = covalent organic framework; SCNC = sulfonated cellulose nanocrystal; N/A = not available. b) Selectivity = proton conductivity / VO²⁺ permeability.

**Table S3. Gaussian fitting results of the WAXS profiles for pristine PFSA and PFSA/MXene composite membranes.**

| peak 0 | | | | | | | |
| --- | --- | --- | --- | --- | --- | --- | --- |
|  | location (Å^-1^) | | FWHM (Å^-1^) | domain spacing (Å) | | crystal size (Å) | |
| PFSA | 0.240 | | 0.054 | 26.167 | | | 116.718 |
| PFSA-0.5M | 0.239 | | 0.054 | 26.311 | | | 115.946 |
| PFSA-1.0M | 0.235 | | 0.054 | 26.756 | | | 116.411 |
| PFSA-1.5M | 0.236 | | 0.053 | 26.599 | | | 118.296 |
| PFSA-2.0M | 0.235 | | 0.057 | 26.694 | | | 109.856 |
| PFSA-3.0M | 0.235 | | 0.058 | 26.740 | | | 108.826 |
| PFSA-5.0M | 0.234 | | 0.061 | 26.838 | | | 103.713 |
|  | | peak 1 | | |  | | |
|  | location (Å^-1^) | | FWHM (Å^-1^) | domain spacing (Å) | | | crystal size (Å) |
| PFSA | 1.171 | | 0.208 | 5.364 | | | 30.218 |
| PFSA-0.5M | 1.169 | | 0.206 | 5.373 | | | 30.416 |
| PFSA-1.0M | 1.169 | | 0.208 | 5.374 | | | 30.249 |
| PFSA-1.5M | 1.167 | | 0.206 | 5.381 | | | 30.426 |
| PFSA-2.0M | 1.165 | | 0.205 | 5.390 | | | 30.636 |
| PFSA-3.0M | 1.165 | | 0.205 | 5.389 | | | 30.585 |
| PFSA-5.0M | 1.163 | | 0.204 | 5.402 | | | 30.726 |
|  | | Peak 2 | | |  | | |
|  | location (Å^-1^) | | FWHM (Å^-1^) | domain spacing (Å) | | | crystal size (Å) |
| PFSA | 2.795 | | 0.380 | 2.247 | | | 16.518 |
| PFSA-0.5M | 2.793 | | 0.381 | 2.249 | | | 16.468 |
| PFSA-1.0M | 2.790 | | 0.382 | 2.251 | | | 16.420 |
| PFSA-1.5M | 2.786 | | 0.380 | 2.254 | | | 16.510 |
| PFSA-2.0M | 2.785 | | 0.383 | 2.255 | | | 16.411 |
| PFSA-3.0M | 2.783 | | 0.379 | 2.256 | | | 16.559 |
| PFSA-5.0M | 2.781 | | 0.379 | 2.258 | | | 16.562 |

Note for Table S3: FWHM means Full Width at Half Maximum.

**Figures**

**Figure S1.** High-resolution XPS spectra of a) O 1s, b) F 1s, and c) C 1s for the few-layer Ti_3_C_2_T_x_.

**Figure S2.** Schematic illustration for the preparation of MXene/PFSA membrane.

**Figure S3.** SEM images of MXene-coated PFSA membranes.

**Figure S4**. a) Cross-sectional SEM images of PFSA/MXene composite membranes with coating thicknesses of 2 μm, 5 μm, and 7 μm; b) proton conductivity and c) hydrogen permeability of the composite membranes as a function of coating thickness.

**Figure S5.** Ion exchange capacity (IEC) of PFSA/MXene composite membranes as a function of MXene content.

**Figure S6.** Schematic diagram of the water contact angle of PFSA membrane and MXene-coated PFSA membranes.

**Figure S7.** a) Tensile strength and b) elongation at break of PFSA membrane and MXene-coated PFSA membranes.

**Figure S8.** a) Solid-state ¹H NMR spectra and b) T_2_ relaxation time distributions of pristine PFSA and PFSA-1.5M composite membranes.

**Figure S9.** Arrhenius plots of proton conductivities.

**Figure S10.** TG analysis of PFSA membrane, MXene-coated PFSA membranes, and MXene.

**Figure S11**. a) UV graphs of VOSO_4_ in 2M H_2_SO_4_ solutions at different concentrations and b) calibration curve for V^4+^ concentrations and absorbance at wavelength ~ 760 nm.

**Figure S12.** a) UV graphs of VOSO_4_ in 2M H_2_SO_4_ solutions at different times for PFSA-1.5M membrane tested by H-type diffusion cell, and b) the relationship between V^4+^ concentrations and diffusion time for membranes.

**Figure S13**. Polarization curves of PFSA membrane and MXene-coated PFSA membranes at 60 ℃ and 80 ℃ in PEMWE.

**Figure S14**. Equivalent circuit model for the Nyquist plot during EIS measurement at 60 ℃ in PEMWE.

**Figure S15.** Fitted R_ct,c_, R_ct,a,_ and R_m_ of a) PFSA and b) PFSA-1.5M membrane from the Nyquist plot under different current densities during EIS test at 60 ℃ in PEMWE.

**Figure S16.** Nyquist plots from in situ EIS measurements under varying current densities at 60 °C for **d**) PFSA membrane and **e**) PFSA-1.5M membranes after durability test of PEMWE.

**Figure S17.** Fitted a)*R_s_*, *R_ct,_* and *R_m_* of b)PFSA and c) PFSA-1.5M membrane from the Nyquist plot under different current densities during EIS test after durability in PEMWE.

**Figure S18.** Ti concentration in the anode outlet water of PEMWE during durability testing, measured by ICP-MS.

**Figure S19.** Cross-sectional SEM images of the PFSA-1.5M membrane after dry-wet cyclic aging tests.

**Figure S20.** a) XPS full spectra and high-resolution XPS spectra of b) C 1s, c) O 1s, and d) F 1s for membranes.

**Figure S21.** High-resolution XPS spectra of Ti 2p for membranes.

References

[1] Oh, K.; Son, B.; Sanetuntikul, J.; Shanmugam, S. Polyoxometalate decorated graphene oxide/sulfonated poly(arylene ether ketone) block copolymer composite membrane for proton exchange membrane fuel cell operating under low relative hu-midity. J. Membr. Sci. 2017, 541, 386–392.

[2] Jin, Y.; Qiao, S.; Costa, J.C.D.; Wood, B.J.; Ladewig, B.P.; Lu, G.Q. Hydrolytically stable phosphorylated hybrid silicas for proton conduction. Adv. Funct. Mater. 2007, 17, 3304–3311.

[3] Ramaswamy, P.; Wong, N.E.; Shimizu, G. MOFs as proton conductors -challenges and opportunities. Chem. Soc. Rev. 2014, 43, 5913–5932.

[4] Colomban, P.; Novak, A. Proton transfer and superionic conductivity in solids and gels. J. Mol. Struct. 1988, 177, 277–308.

[5] G. Kresse, J. Furthmüller, Efficiency of Ab initio total energy calculations for metals and semiconductors using a plane-wave basis set, Comput. Mater. Sci.,1996, 1, 15−50.

[6] G. Kresse, J. Furthmüller, Efficiency iterative schemes for Ab initio total-energy calculations using a plane-wave basis set, Phys. Rev. B.,1996, 16, 11169−11186.

[7] J.P. Perdew, K. Burke, M. Ernzerhof, Generalized gradient approximation made simple, Phys. Rev. Lett.,1996, 18, 3865−3868.

[8] H. J. Monkhorst, J. D. Pack, Special point for Brillouin-zone integrations, Phys. Rev. B., 1976, 13, 5188−5192.

[9] P. E. Blöchl, Projector augmented-wave method, Phys. Rev. B.,1994, 50, 17953−17979.

[10] G. Henkelman, B. P. Uberuaga and H. Jónsson, A climbing image nudged elastic band method for finding saddle points and minimum energy paths, J. Chem. Phys., 2000, 113, 9901−9904.

[11] J. Sun, D. Shi, H. Zhong, X. Li, H. Zhang, Investigations on the self-discharge process in vanadium flow battery. J. Power Sources, 2015, 294, 562−568.

[12] L. Zhu, H. Yang, T. Xu, L. Wang, J. Lei, C. Si, Engineered Nanochannels in MXene Heterogeneous Proton Exchange Membranes Mediated by Cellulose Nanofiber/Sodium Alginate Dual Crosslinked Networks. Adv. Funct. Mater. 2024, 35.

[13] M. Huang, A. Song, X. Ben, W. Ji, Y. Pan, H. Rao, Self-Humidifying and Super-Protonic Conductivity of SPEEK-Based Composite Proton Exchange Membranes Incorporated by Functionalized MXene and Modified TiO₂ Nanofillers. Nanomaterials 2026, 16.

[14] P. Waribam, K. Jaiyen, C. Samart, M. Ogawa, G. Guan, S. Kongparakul, MXene-Copper Oxide/Sulfonated Polyether Ether Ketone as a Hybrid Composite Proton Exchange Membrane in Electrochemical Water Electrolysis. Catal. Today 2023, 407, 96-106.

[15] Y. Wang, J. Zhao, L. Li, Y. Zhang, Y. Wang, X. Gao, B. Su, F. Li, X. Wang, L. Shi, et al., Study on Sulfonated MXene/Halloysite Nanotube Composite-Modified SPI Proton Exchange Membrane: Proton Transport Mechanism and Durability Enhancement. J. Mater. Sci. 2026, 61, 22710-22728.

[16] P. Guan, J. Lei, Y. Zou, Y. Zhang, Improved Thermo-Mechanical Properties and Reduced Hydrogen Permeation of Short Side-Chain Perfluorosulfonic Acid Membranes Doped with Ti₃C₂Tₓ. Materials 2021, 14, 7875.

[17] S. Zhao, R. Wang, T. Tian, H. Liu, H. Zhang, H. Tang, Self-Assembly-Cooperating in Situ Construction of MXene–CeO_2_ as Hybrid Membrane Coating for Durable and High-Performance Proton Exchange Membrane Fuel Cell. ACS Sustainable Chem. Eng. 2022, 10, 4269-4278.

[18] X. Jia, Z. Lu, K. Huai, X. Yuan, Z. Yang, R. Huang, S. Zhai, Q. Ye, Z. Deng, J. Lin, et al., Covalent Organic Framework In-Situ Grown on MXenes to Improve Ion Selectivity of Composite Membranes for Vanadium Flow Battery. J. Power Sources 2025, 637.

[19] X. Jia, K. He, X. Liu, Z. Yang, R. Huang, Y. Liu, J. Lin, X. Liu, J. Wang, S. He, Highly Selective Proton Exchange Membrane Enabled by Organically Modified MXene for Vanadium Flow Battery. J. Polym. Sci. 2025, 63, 5094-5105.

[20] Y. Zhang, D. Zhang, Q. Zhou, X. Zhang, S. Hou, H. He, X. Wu, Q. Li, J. Liu, C. Yan, Interfacial Engineering of MXene‐Based Heterogeneous Membranes with Enhanced Ion Selectivity in Vanadium Redox Flow Battery. Adv. Funct. Mater. 2025, 36.

[21] W. Zhong, L. Lv, Z. Wang, Z. Wang, Fabrication of Sulfonated Cellulose Nanocrystal/MXene Hybrid Proton Exchange Membrane and Its Synergistic Effect in Vanadium Redox Flow Battery. Nano Lett. 2025, 25, 4339-4346.
